# Survival time in severe hemorrhagic shock after perioperative hemodilution is longer with PEG-conjugated human serum albumin than with HES 130/0.4: a microvascular perspective

**DOI:** 10.1186/cc6874

**Published:** 2008-04-18

**Authors:** Judith Martini, Pedro Cabrales, Ananda K, Seetharama A Acharya, Marcos Intaglietta, Amy G Tsai

**Affiliations:** 1 Department of Bioengineering, University of California, San Diego, Gilman Dr, La Jolla, California 92093, USA; 2 La Jolla Bioengineering Institute, Coast Blvd South, La Jolla, California 92037, USA; 3 Department of Hematology and Medicine, Albert Einstein College of Medicine, Morris Park Avenue, Bronx, New York 10461, USA

## Abstract

**Introduction:**

Preoperative hemodilution is an established practice that is applied to reduce surgical blood loss. It has been proposed that polyethylene glycol (PEG) surface decorated proteins such as PEG-conjugated human serum albumin may be used as non-oxygen-carrying plasma expanders. The purpose of this study was to determine whether there is any difference in survival time after severe hemorrhagic shock following extreme hemodilution using a conventional hydroxyethyl starch (HES)-based plasma expander or PEG-albumin.

**Methods:**

Experiments were performed using the hamster skinfold window preparation. Human serum albumin that was surface decorated with PEG was compared with Voluven 6% (Fresenius Kabi, Austria; a starch solution that is of low molecular weight and has a low degree of substitution; HES). These plasma expanders were used for a 50% (blood volume) exchange transfusion to simulate preoperative hemodilution. Exchange transfusion was followed by a 60% (blood volume) hemorrhage to reproduce a severe surgical bleed over a 1 hour period. Observation of the animal was continued for another hour during the shock phase.

**Results:**

The PEG-albumin group exhibited significantly greater survival rate than did the HES group, in which none of the animals survived the hemorrhage phase of the experiment. Among the treatment groups there were no changes in mean arterial pressure and heart rate from baseline after hemodilution. Both groups experienced gradual increases in arterial oxygen tension and disturbance in acid-base balance, but this response was more pronounced in the HES group during the shock period. Mean arterial pressure remained elevated after the initial hemorrhage period in the PEG-albumin group but not in the HES group. Maintenance of a greater mean arterial pressure during the initial stages of hemorrhage is proposed to be in part due to the improved volume expansion with PEG-albumin, as indicated by the significant decrease in systemic hematocrit compared with the HES group. PEG-albumin treatment yielded higher functional capillary density during the initial stages of hemorrhage as compared with HES treatment.

**Conclusion:**

The ability of PEG-albumin to prolong maintenance of microvascular function better than HES is a finding that would be significant in a clinical setting involving preoperative blood management and extreme blood loss.

## Introduction

Preoperative blood management techniques are increasingly being standardized, with the aim being to limit allogenic blood transfusions in elective surgery [1,2]. Surgical patients who are managed in accordance with bloodless or limited blood usage standards are hemodiluted with a colloid solution before surgery in order to salvage autologous blood for later use. This procedure results in a moderate reduction in hematocrit but, because of compensatory increases in cardiac output, there is no adverse effect on oxygen delivery [3]. The success of this procedure significantly depends on achieving and maintaining normovolemic status.

Conventional colloids such as dextrans, gelatins, hydroxyethyl starch (HES), and albumin have been used for blood volume replacement therapy and have become well established for preoperative volume substitution [4,5]. However, which colloid to use remains a point of contention because, in addition to their intravascular volume expansion properties, most of these materials also have a variable influence on other factors such as coagulation and renal function.

Colloids provide lasting circulating volume expansion because of their oncotic properties and slow clearance rate from the circulation [6,7]. HES, a natural polymer of amylopectin, has a molecular structure that allows for a variety of chemical modifications, which result in different degrees of volume expansion and half-life properties, depending on the degree of substitution or hydroxylation, molecular weight and concentration [4,5].

It has been proposed that polyethylene glycol (PEG) surface decorated proteins such as PEG-conjugated human serum albumin [8] and hemoglobin [9,10] may represent new types of plasma expander: non-oxygen-carrying and oxygen-carrying, respectively. PEGylation of these proteins yields oncotic properties similar to those of the natural protein but at much lower concentrations. Therefore, less PEG-albumin is needed to attain the same oncotic effect as its counterpart protein. Animal studies provide evidence that PEG-albumin (carrying six copies of PEG-5,000 chains per molecule), at concentrations of 4 g albumin/dl, is an effective plasma expander during hemodilution [11,12] and resuscitation fluid for use in hemorrhagic shock [13]. At concentrations as low as 2.5 g albumin/dl, PEG-albumin (carrying 10 copies of PEG-5,000 chains per molecule) is better able to resuscitate from induced endotoxemia, thus preventing the development of circulatory collapse, as compared with 6 g/dl dextran 70 (molecular weight 70 kDa) [14]. PEG-albumin has the advantages of a longer half-life because of reduced glomerular filtration and diminished proteolysis [15,16]. Additionally, PEGylation reduces the potential immunologic activity [17] and drug-binding capacity [18] of albumin. In the present study we use a new type of PEG-albumin, in which human serum albumin (HSA) is surface decorated with about six PEG-5,000 chains through extension arm facilitated PEGylation. This new type of molecule could be suitable as a plasma expander, which is effective at reduced plasma concentrations and potentially has a better defined pharmacokinetic profile because of its uniform molecular size.

A critical component of blood volume replacement fluids is their plasma expansion properties, and how these properties promote the maintenance of systemic and microvascular function during extreme blood volume challenges. In this study we tested the functionality of PEG-albumin used experimentally in preoperative hemodilution (50% blood volume) followed by a significant surgical bleed (60% exponential bleed). Investigations were conducted at the microvascular level in the hamster window chamber model hemodiluted with Voluven (Fresenius Kabi, Graz, Austria; HES). Results were compared with PEG-albumin using the same protocol. The objective was to determine the relative merits of these colloids as preoperative hemodilution plasma expanders, and to determine whether there was any effect on survival time and maintenance of microvascular perfusion after 1 hour of hemorrhage.

## Materials and methods

### Animal preparation

Investigations were conducted in male golden Syrian hamsters weighing 50 to 65 g (Charles River Laboratories, Boston, MA, USA). Animal handling and care were provided following the procedures outlined in the Guide for the Care and Use of Laboratory Animals (US National Research Council, 1996). The local Animal Subjects Committee approved the study. The hamster window chamber model is widely used for microvascular studies in the unanesthetized state, and the complete surgical technique for the preparation has previously been described in detail [19,20]. The animal was allowed at least 2 days for recovery; its chamber was then assessed under the microscope for any signs of edema, bleeding, or unusual neovascularization. Barring these complications, the animal was anesthetized again with pentobarbital sodium. Arterial and venous catheters (polyethylene-50) were implanted in the carotid artery and jugular vein, respectively. Catheters were tunneled under the skin and exteriorized at the dorsal side of the neck, where they were attached to the chamber frame with tape. This model allows the study of an intact subcutaneous tissue and a single thin retractor muscle free from surgical manipulation and exposure to ambient atmospheric conditions.

### Inclusion criteria

Animals were deemed suitable for the experiments if the following were satisfied: systemic parameters were within normal range, namely heart rate (HR) above 320 beats/minute, mean arterial blood pressure (MAP) above 80 mmHg, systemic hematocrit above 45%, and arterial oxygen tension (PaO_2_) above 50 mmHg; and microscopic examination of the tissue under high magnification (40 × objective; NA [numerical aperture] 0.7 SW [salt water]; Olympus, Central Valley, PA, USA) did not reveal signs of edema or bleeding.

### Systemic parameters

MAP and HR were monitored continuously (MP 150; Biopac Systems, Inc., Santa Barbara, CA, USA), except when the catheters were used to take samples for laboratory parameters. Arterial blood samples taken in heparinized microcapillary tubes (40 μl) were centrifuged to determine hematocrit.

### Blood chemistry

Arterial blood was collected in heparinized glass capillaries (0.05 ml) from the carotid catheter and immediately analyzed for PaO_2_, arterial carbon dioxide tension (PaCO_2_), and pH (Blood Chemistry Analyzer 248; Bayer, Norwood, MA, ASA). The comparatively low PaO_2 _and high PaCO_2 _of these animals was a consequence of their adaptation to a fossorial environment.

### Microhemodynamics

Arteriolar and venular blood flow center line velocities were measured online using the photodiode cross-correlation method [21] (Photo Diode/Velocity Tracker 102B; Vista Electronics, San Diego, CA, USA). Measured centerline velocity (*V*) was corrected according to vessel size to obtain mean red blood cell (RBC) velocity from centerline velocity measurements [22]. Video image shearing was used to measure vessel diameter (*D*; Image Shearing Monitor; Vista Electronics, San Diego, CA, USA) [23]. Blood flow (*Q*) was calculated as *Q *= *V *× π (*D*/*2*)^2^. Changes in arteriolar and venular diameter from baseline were used as indicators of changes in vascular tone.

### Functional capillary density

Capillaries were considered functional if RBC transit was observed through the capillary segments during a 30-second period. Functional capillary density (FCD) was tabulated from capillary lengths with RBC transit in an area comprised of 20 successive microscopic fields under 40× magnification. FCD (cm^-1^) is the total length of RBC-perfused capillaries divided by the surface area.

### Experimental design

The unanesthetized animal was placed into a restraining tube for the duration of the experiment. The tube containing the conscious animal was fixed to the microscope stage of an intravital microscope (BX51 W1, 40× objective, NA 0.7 SW; Olympus). The tissue image was projected onto a CCD camera (4815-2000; COHU, San Diego, CA, USA) connected to a timer and viewed on a closed circuit monitor. Arterioles and venules, chosen by their visual acuity (three to seven of each type), were characterized by their blood flow, velocity and diameter. FCD was assessed. Vessels chosen for baseline observations were followed throughout the experiment to eliminate bias. Animals were allowed 30 minutes to adjust to the tube environment before measuring baseline parameters (MAP, HR, arterial blood gases and pH, and systemic hematocrit).

### Isovolemic hemodilution

An isovolemic hemodilution of 50% blood volume (BV; estimated as 7% of body weight) was performed by simultaneous withdrawal of blood from the arterial catheter and infusion of the study solution into the venous catheter at a rate of 0.1 ml/minute (33 pump; Harvard Apparatus, Hollister, MA, USA).

### 60% Blood volume exponential hemorrhage and shock

The animals were hemorrhaged (60% of BV) 10 minutes after the completion of the hemodilution during a 1-hour period at a rate of 0.3 ml/minute. Arterial blood was removed by a peristaltic pump (P720; Instech, Plymouth Meeting, PA, USA) connected to the arterial line. The pump was started at the beginning of each 10-minute period and run for a time calculated to complete removal of 60% of the blood volume by the end of 1 hour. The total blood volume (TBV) at the end of each 10-minute period is as follows:

TBV = TBV0 × e^-0.01526*t*^

Where TBV0 is the initial blood volume (assumed to be 70 ml/kg) and *t *is time (minutes). The amount of blood withdrawal each time was determined from this algorithm [24], and therefore we drew progressively smaller amounts of blood during the hour to simulate a surgical bleed. At the end of the 60-minute hemorrhage period, the animals were monitored for an additional 1-hour period of shock before they were killed euthanasia. The animals were categorized as nonsurvivors and killed earlier if at any time during the protocol their MAP fell below 30 mmHg for more than 10 minutes.

Systemic and microvascular parameters were measured at baseline and after hemodilution, hemorrhage and shock. MAP, HR, and FCD were measured every 10 minutes during the hemorrhage period after each blood withdrawal. Microvascular vessel diameter and blood flow were measured at 30-minute intervals after the first hemorrhage (H30 and H60) and continued in the shock phase (S30 and S60). Arterial blood gases and hematocrit were measured at baseline, after hemodilution, and the end of the shock period.

### Study materials

Table 1 presents the physical properties of the study solutions PEG-albumin and HES.

**Table 1:** Properties of the study solutions

Property	Hamster blood	PEG-albumin	HES
Concentration (%)	-	4	6
Average molecular weight (kDa)		96	130
Suspending fluid	-	Phosphate buffer	Saline
Viscosity (cp)	4.2	2.2	2.1
COP (mmHg)	16	42	42
Degree of substitution	-	-	0.40

Shown are the properties of the study solutions and of hamster blood. The concentrations of the fluids were chosen so that their viscosity and colloid osmotic pressures (COPs) were matched. To achieve a match of these parameters, the colloids were used at different concentrations. HES, hydroxyethyl starch; PEG-albumin, polyethylene glycol-conjugated human serum albumin.

#### Polyethlyene surface decorated human serum albumin (PEG-albumin)

PEG-albumin is synthesized by extension arm facilitated PEGylation protocol using lyophilized and essentially fatty acid free, approximately 99% pure human serum albumin (HSA; Sigma-Aldrich, Inc., MO, USA), 2-iminothiolane (lot #10222; BioAffinity Systems, Inc. Rockford, IL, USA), and maleimide phenyl PEG 5000 (lot #01186; BioAffinity Systems). Using extension arm facilitated PEGylation, the HSA was surface decorated with an average of six copies of PEG-5,000 [25]. Human serum albumin in phosphate-buffered saline (pH 7.4) at a concentration of 32 mg/ml was incubated with 0.69 mg/ml of 2-iminothiolane (10-fold molar excess over albumin) and 50 mg/ml maleimide phenyl PEG-5,000 (20-fold molar excess over albumin) for an overnight reaction under cold conditions (4°C). The ratio of HSA and iminothiolane was standardized such that an average of six free thiols generated on the HSA, which are estimated using the 4-PDS method (4,4'-dithiodipyridine; Sigma-Aldrich, St. Louis, MO, USA) [26]. All of the reaction components were mixed in a single step so that thiols generated on the protein *in situ *are immediately modified by maleimide phenyl PEG-5,000. Excess iminothiolane and PEG reagent present in the reaction mixture was removed by tangential flow filtration through 50 kDa molecular weight cutoff membranes against phosphate-buffered saline (pH 7.4) using the Minim™ Tangential Flow Filtration instrument (Pall Corporation, Ann Arbor, MI, USA). After complete removal of unbound PEG (established by size exclusion chromatography and monitoring of the refractive index of the effluent) the reaction mixture was concentrated to 40 mg/ml. PEGylated human serum albumin sample was sterilized by filtering through 0.22 μ Millipore filters. The concentration of PEGylated HSA was verified using the Bradford protein assay (Pierce Biotechnology, Inc. Rockford, IL, USA). Measurement of colloidal oncotic pressure at room temperature was about 42 mmHg and viscosity 2.2 cP at 37°C for 4% solution. The number of PEG chains on HSA molecule was analyzed by nuclear magnetic resonance and mass spectroscopy, which has confirmed attachment of an average of six copies PEG-5,000 chains per HSA molecule. Based on SDS-PAGE, nuclear magnetic resonance, and MALDI-TOF-MS (matrix assisted laser desorption ionisation time-of-flight mass spectrometry), the average molecular weight of this hexaPEGylated HSA is about 96 kDa. High-performance liquid chromatography analysis showed the product to have one broad peak with slight assymmetry. This peak position corresponds to the HSA molecule with six PEG chains, with a small contribution from HSA molecule with five PEG chains. Hydrodynamic radius of the hexaPEGylated HSA is at the range of 7.2 to 7.8 nm. The availability of the free thiols on HSA after PEGylation was also estimated using the 4-PDS method and is about 0.1 group per molecule. It is assumed that the molecular radius of this product is around 7.5 nm, as compared with 4 nm for the HSA.

#### Hydroxyethyl starch

HES of low molecular weight and with low degree of substitution (mean molecular weight 130 ± 20 kDa, degree of substitution 0.4) was formulated at 6% (weight/vol) in 0.9% saline injection (Voluven; Fresenius-Kabi) [27].

### Statistical methods

One way analysis of variance was performed between time points of interest within a treatment group, with Tukey *post hoc *analysis when differences were found; this method that accounts for the progressive decrease in number of observations resulting from loss of animals. Mann-Whitney test was used to compare the two treatment group at time points of interest. The product limit method (Kaplan-Meier) was used to produce survival curves, and analysis of survival was conducted using the log-rank test (Mantel-Cox). Statistical analyses were performed with Prism 5.01 software (Graphpad, San Diego, CA, USA). Results were considered statistically significantly different at P < 0.05. Data are presented as mean ± standard deviation (with the exception of flow, which is presented as mean ± standard error of the mean).

## Results

Ten animals were entered into the study and divided randomly into two treatment groups before the experiment: PEG-albumin (n = 5) and HES (n = 5). Systemic data from baseline for both groups were combined because there were no differences between groups.

### Survival

**Figure 1:**
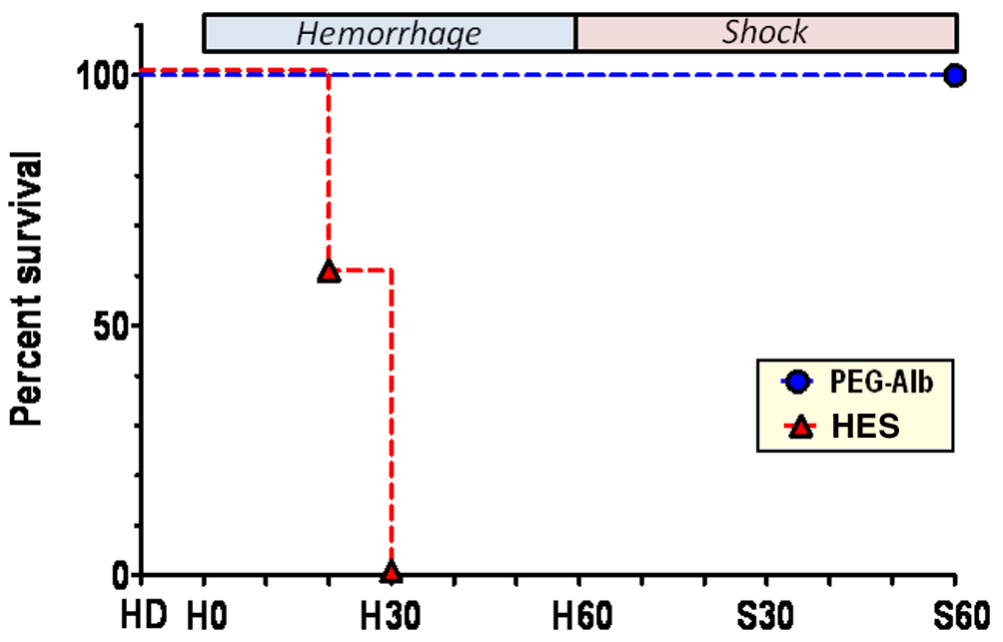
Percentage survival of the different treatment groups during the protocol after the hemodilution. Treatment groups: polyethylene glycol-conjugated human serum albumin (PEG-albumin; solid black circles) and hydroxyethyl starch (HES; solid black triangles).

Figure 1 shows the percentage survival during the experiment. All animals in the PEG-albumin group survived the protocol whereas none of the animals in the HES group completed the hemorrhage phase of the experimental protocol. The difference in survival between PEG-albumin and HES was statistically significant (*P *= 0.003).

### Systemic and laboratory parameters

MAP and HR during the experimental protocol after hemodilution (HD) and at H0, H30, H60, S30 and S60 are presented in Table 2. These time points were chosen for comparison because they represent the most significant events in this experimental protocol: HD, hemodilution, H0 is immediately after the first and most extreme hemorrhage; H30 is the midpoint in the hemorrhage period, when most of the 60% volume has been withdrawn; H60 is the end of the hemorrhage period/start of the shock period; S30 is 30 minutes into shock; and S60 is the end of the experiment. Hemodilution did not significantly change MAP and HR among groups relative to baseline because the procedure was performed at a slow rate, in order to allow the animals to compensate for the lowered oxygen carrying capacity and changes in blood rheology. Hemorrhage significantly reduced the MAP and HR in both treatment groups, with PEG-albumin being able to maintain a statistically higher MAP compared with HES until 30 minutes into the hemorrhage.

**Table 2:** Mean arterial pressure and heart rate

	PEG-albumin		HES	
	
	MAP	HR	MAP	HR
HD	96.6 ± 5.6	482.4 ± 28.8	100.0 ± 8.3	493.8 ± 20.9
H0	56.8 ± 7.2*^†b^	396.8 ± 65.0*^†^	43.0 ± 10.7*^†^	305.3 ± 83.5*^†^
H30	43.0 ± 7.6*^†b^	342.8 ± 54.0*^†^	33.3 ± 6.7*^†^	376.8 ± 98.8*^†^
H60	42.6 ± 10.3*^†^	367.2 ± 34.6*^†^	-	-
S30	46.0 ± 10.2*^†^	338.2 ± 18.5*^†^	-	-
S60	46.0 ± 11.9*^†^	325.8 ± 21.0*^†^	-	-

Shown are the changes in MAP and HR between the two study groups at different time points. Baseline: MAP (mmHg) = 107.4 ± 8.2, HR (beats/minute) = 466.3 ± 24.2. Within the same treatment group: *versus baseline; ^†^versus hemodilution. Among treatments: PEG-albumin versus ^b^HES. HD, hemodilution; H0, beginning of hemorrhage; H30, midpoint in the hemorrhage period when most of the 60% volume has been withdrawn; H60, end of the hemorrhage period; HES, hydroxyethyl starch; HR, heart rate; MAP, mean arterial pressure; PEG-albumin, polyethylene glycol-conjugated human serum albumin; S30, 30 minutes after beginning of shock; S60, end of the experiment.

Laboratory parameter changes are presented in Table 3. As expected, hemodilution reduced hematocrit. The PEG-albumin group had a hematocrit that was statistically lower than that in the HES group. Lower PaO_2 _values were obtained for the PEG-albumin versus HES. As expected, at the end of the hemorrhage phase and during the shock phase, there was a progressive increase in PaO_2_, and a decrease in PaCO_2 _and pH (H60 to S60). The HES group did not compensate for hemorrhage and consequently had a reduced albeit positive acid-base balance after hemodilution.

**Table 3:** Hemoglobin, hematocrit and arterial blood gases

	Baseline	HD		H60		S60	
		
		PEG-albumin	HES	PEG-albumin	HES	PEG-albumin	HES
Hematocrit (%)	46.4 ± 1.4	26.4 ± 0.9*^b^	31.6 ± 1.1*	18.2 ± 0.4*^†^	-	18.1 ± 0.5*^†^	-
PaO_2 _(mmHg)	58.9 ± 8.7	59.5 ± 11.3^b^	69.6 ± 7.0	100.0 ± 12.8*^†^	-	121.0 ± 14.3*^†‡^	-
PaCO_2 _(mmHg)	59.1 ± 10.1	59.7 ± 5.0	51.1 ± 5.3	45.3 ± 12.9	-	36.1 ± 8.1*^†^	-
pH arterial	7.388 ± 0.030	7.387 ± 0.036	7.349 ± 0.025	7.251 ± 0.076*^†^	-	7.109 ± 0.047*^†‡^	-
BE (mmol/l)	5.5 ± 1.9	5.3 ± 2.3	1.8 ± 1.8*	-8.2 ± 2.8*^†^	-	-18.0 ± 4.7*^†^	-

Shown are changes in hemoglobin, hematocrit and arterial blood gases between the two study groups at different time points. Within the same treatment group: *versus baseline; ^†^versus HD; ^‡^versus H60. Among treatments: PEG-albumin versus ^b^HES. BE, base excess; HD, hemodilution; H60, end of the hemorrhage period; HES, hydroxyethyl starch; PaO_2_, arterial oxygen tension; PaCO_2_, arterial carbon dioxide tension; PEG-albumin, polyethylene glycol-conjugated human serum albumin; S60, end of the experiment.

### Microhemodynamics

**Figure 2:**
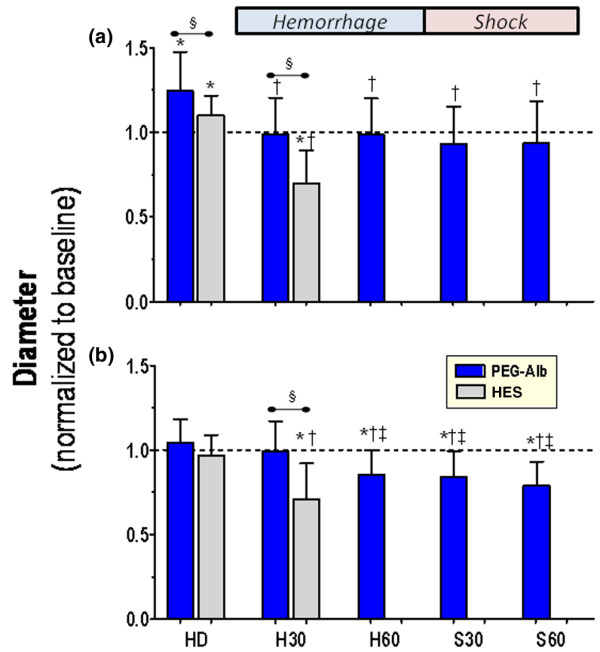
Arteriolar and venular diameters. Changes to **(a) **arteriolar and **(b) **venular diameters at each time point of interest. Analysis within the same treatment group: *P < 0.05 relative to baseline; ^†^*P *< 0.05 relative to HD, ^‡^*P *< 0.05 relative to H30. Analysis between treatments at the same time point (denoted by the horizontal bar): ^§^*P *< 0.05. Data are expressed as mean ± standard deviation. HD, hemodilution; H0, beginning of hemorrhage; H30, midpoint in the hemorrhage period when most of the 60% volume has been withdrawn; H60, end of the hemorrhage period; S30, 30 minutes after beginning of shock; S60, end of the experiment.

**Figure 3:**
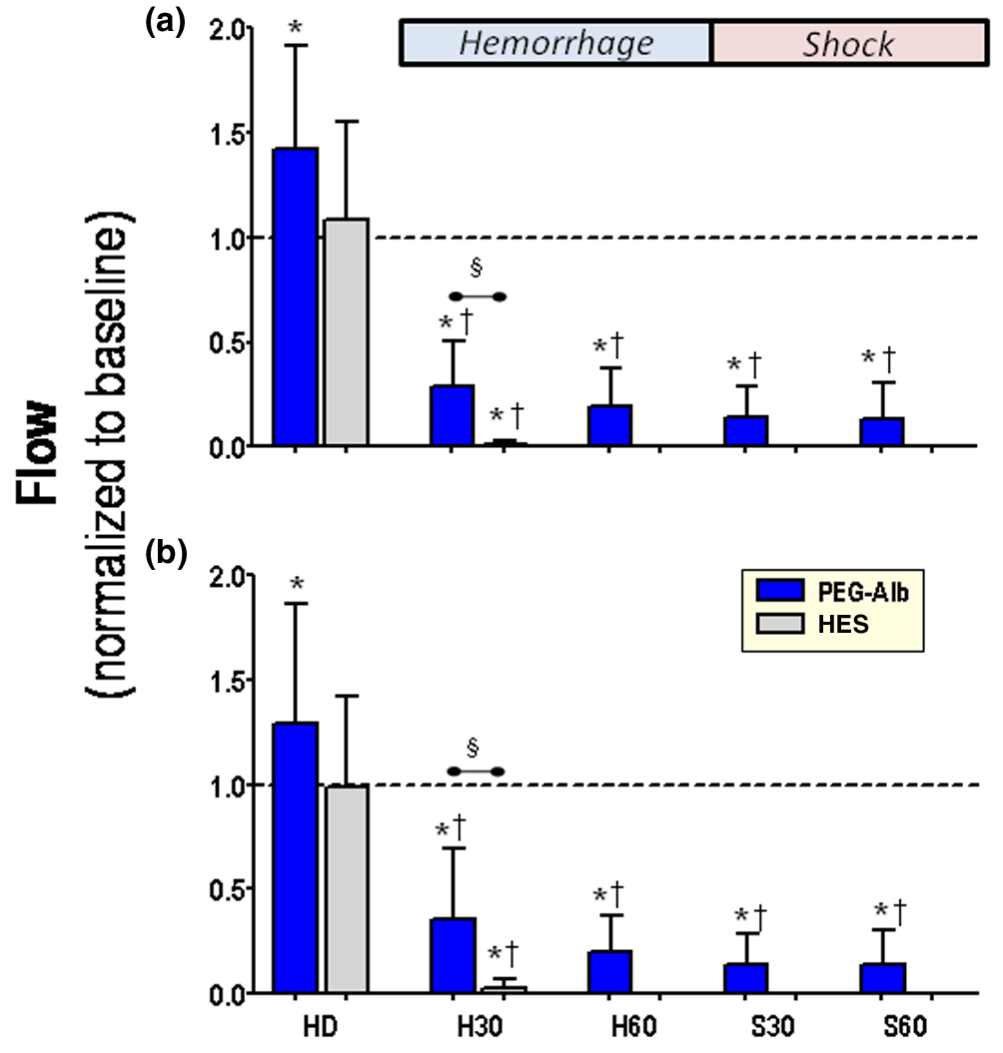
Arteriolar and venular blood flow. Changes to **(a) **arteriolar and **(b) **venular blood flow at each time point of interest. Analysis within the same treatment group: *P < 0.05 relative to baseline; ^†^*P *< 0.05 relative to HD, ^‡^*P *< 0.05 relative to H30. Analysis between treatments at the same time point (denoted by the horizontal bar): ^§^*P *< 0.05. Data are presented mean ± standard error of the mean. HD, hemodilution; H0, beginning of hemorrhage; H30, midpoint in the hemorrhage period when most of the 60% volume has been withdrawn; H60, end of the hemorrhage period; S30, 30 minutes after beginning of shock; S60, end of the experiment.

Figures 2 and 3 present the changes in diameter and blood flow for arterioles and venules during the experiment.

#### Arteriolar diameter and flow

Hemodilution with all solutions resulted in statistically significant arteriolar vasodilation. The PEG-albumin group exhibited a greater vasodilatory response than did the HES group. During the hemorrhage and shock phases, the dilated arterioles for the PEG-albumin group vasoconstricted back to baseline levels. This response by the PEG-albumin animals was maintained for the entire observation period and was statistically different from that in HES animals. The HES animals exhibited significant arteriolar vasoconstriction relative to baseline during hemorrhage.

Arteriolar vasodilation after hemodilution with PEG-albumin was concomitant with a significant increase in blood flow, whereas the flow with HES remained unchanged from baseline. After the initial hemorrhage step, both groups experienced reduced blood flow relative to baseline and hemodilution. At the H30 time point, the HES-treated group had a statistically significant reduction in flow when compared with the PEG-albumin group.

#### Venular diameter and flow

Hemodilution did not affect venular diameter, which remained unchanged during the hemorrhage phase relative to baseline in the PEG-albumin group. The HES group had venular vasoconstriction, which was significant relative to baseline and hemodilution, and the other study group at these time points. After the exponential hemorrhage was completed and the animals continued into the shock phase (60% of the initial blood volume was removed [H60]) the venular vessels vasoconstricted at all time points relative to baseline.

Blood flow was increased in the PEG-albumin group after hemodilution but remained unchanged relative to baseline in the HES group. At the H30 time point all animals in the HES treatment group had pressures that categorized them as 'nonsurvivors' and had essentially no microvascular perfusion.

#### Functional capillary density

**Figure 4:**
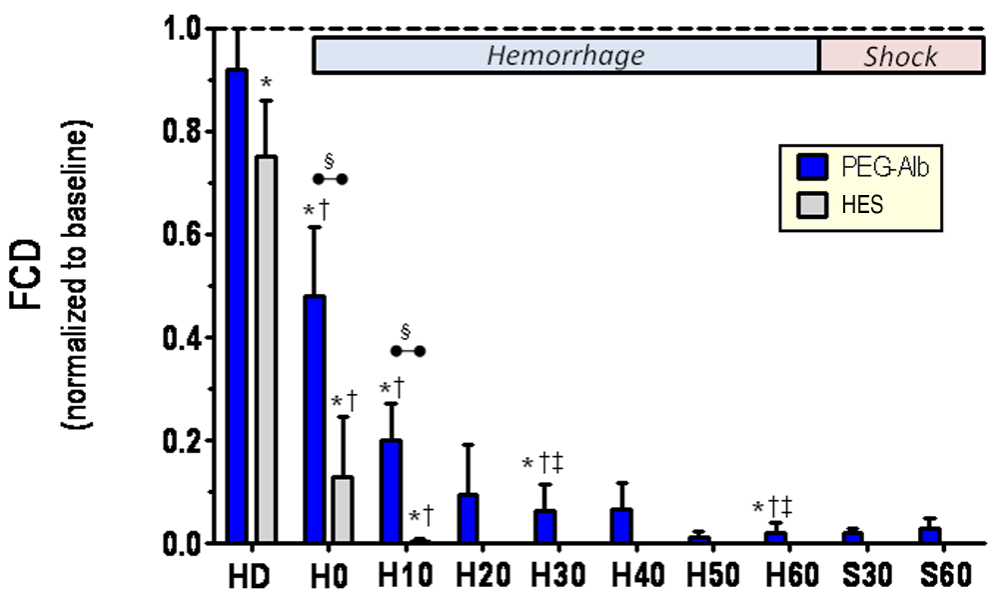
FCD after hemodilution and during hemorrhage (H0 to H60). The functional capillary density (FCD) level at the end of hemorrhage (H60) was unchanged during the shock period, S30 and S60. Data are presented mean ± standard deviation. Analysis within the same treatment group: *P < 0.05 relative to baseline; ^†^*P *< 0.05 relative to HD, ^‡^*P *< 0.05 relative to H30. Analysis between treatments at the same time point (denoted by the horizontal bar): ^§^*P *< 0.05.

Changes in FCD after hemodilution and during the hemorrhage phase of the experiment are shown in Figure 4. The FCD data were evaluated at time points hemodilution, H0, H10, H30 and H60, the other time points are shown to illustrate the trend in the data. Hemodilution caused a significant fall in FCD for the HES group, but the PEG-albumin group remained at levels not different from baseline. Immediately after the largest blood volume withdrawal (at H0), FCD was significantly reduced relative to baseline and hemodilution. However, PEG-albumin was able to sustain a higher FCD level relative to HES. A similar pattern was observed at H10. FCD dropped as low as 0.08 of baseline during later time points, a level that was maintained during shock phase.

## Discussion

The principal finding of this study is the notable difference in outcome between using PEG-albumin and HES solutions in a protocol designed to simulate a preoperative hemodilution followed by significant surgical hemorrhage (exponential bleed of 60% BV). All PEG-albumin treated animals survived hemorrhage and completed the study, whereas HES-treated animals did not even survive the hemorrhage period. A factor related to the survival rates was the significantly higher FCD obtained with the PEG-albumin group as compared with the other group after the initial hemorrhage.

Previous hemorrhagic shock studies conducted in our laboratory demonstrated the importance of maintaining tissue perfusion and demonstrated a strong correlation between FCD and survival [28]. Local perfusion probably limits metabolite accumulation during low flow states [13], and this may lead to better outcomes [28].

Even though FCD was maintained in surviving animals during the initial hemorrhage phase, it later dropped to levels as low as 0.08 compared with baseline because of the severity of the protocol.

The hematocrit differences between groups after hemodilution could be indicative of differences in intravascular volume. Volume expansion beyond baseline blood volume after the hemodilution would preload the animal with fluids and may affect outcome. At the microvascular level, PEG-albumin achieved higher blood flow and FCD in comparison with HES. Because the period between hemodilution and initiation of hemorrhage was 10 minutes, it is likely that only volume expansion and not volume retention plays a role in outcome for this study.

The hemodilution in the present study reduced the oxygen reserve, as indicated by the decreases in systemic hematocrit to 31.6% and 26.4% for HES and PEG-albumin, respectively. In a resting animal, this hemodilution level corresponds to a reduction in oxygen-carrying capacity that has been shown not to affect oxygen delivery or tissue oxygenation [29]. In the HES group, which exhibited greater oxygen-carrying capacity after hemodilution as compared with the PEG-albumin group, the PaO_2 _increased; this suggests a compensatory hyperventilation response, which was not present with the PEG-albumin group.

The effects of the suspending fluid of these solutions can also influence outcome. The clinical relevance of hyperchloremic acidosis is not fully understood and remains controversial [30]. HES is suspended in saline, and when it is administered in large volumes intravenously – as in the case of hemodilution – this could lead to a nonphysiologic chloride load and metabolic acidosis, as suggested by the observed disturbance in acid-base balance. The HES group exhibited reduced pH and base excess. Studies in both animals and humans show a relationship between electrolyte balanced and nonbalanced solutions in terms of the extent of tissue perfusion [31,32]. Therefore PEG-albumin formulated in a phosphate buffer at a physiological pH may perform better in terms of sustaining flow and FCD.

Plasma expander viscosity has been shown to influence FCD and local tissue perfusion during extreme hemodilution [33]. Increasing blood viscosity during hemodilution by using a high-viscosity plasma expander (6% dextran 500 kDa, 0.7% alginate) leads to better microvascular perfusion in comparison with using a low-viscosity plasma expander (6% dextran 70 kDa) [29,34,35]. The viscous drag exerted by plasma expanders is proposed to interact with the endothelium and trigger a vasodilator response. If the interaction is significantly reduced, as in the case of low viscosity plasma expanders in extreme hemodilution, then the production of vasodilators such as nitric oxide by the endothelium is reduced)[36]. Therefore, viscosity of the study fluids could influence the results obtained after hemodilution. Studies of PEG-albumin in extreme hemodilution have shown it to be effective at sustaining high levels of microvascular perfusion, even though it is only a moderately highly viscogenic fluid [11]. In the case of PEG-albumin, the mechanism related to viscosity remains unclear and it has been hypothesized that it may activate endothelial pathways by direct physical interaction of the PEG with the glycocalyx on the endothelium surface [37].

Nitric oxide plays a role in vascular regulation. Therefore the ability of albumin to transport nitric oxide as S-nitrosothiols on its surface could alter the vascular distribution of nitric oxide and in turn regulate local blood flow [38]. PEGylation of albumin adds pseudo-thiols onto the protein surface, complementing the number of natural thiols already available for nitric oxide transport. This attribute of PEG-albumin may partially account for its ability to maintain microvascular perfusion and FCD above other colloids [11] without significantly increasing plasma viscosity above normal. During hemorrhage, RBC loss reduces the nitric oxide scavenging potential of blood, whereas ischemia induces nitric oxide synthase. Under these conditions, the excess of nitric oxide in blood can be taken up by PEG-albumin to be redistributed and then released in hypoxic or acidic tissues. PEG-albumin has been shown to be an effective resuscitation fluid compared with other colloidal plasma expenders in various hemorrhagic shock scenarios [13,39-41]. Although the major component of recovering local perfusion from hemorrhagic shock is the ability of the plasma expander to recover and sustain blood volume, a beneficial effect of PEG-albumin could be related to the transport and redistribution of nitric oxide, leading to improved local flow by promoting vasodilation, reduced leukocyte adhesion and platelet aggregation [42].

## Conclusion

In the present study we tested the functionality of PEG-albumin used experimentally in preoperative hemodilution followed by a significant surgical bleed. Survival time was longer with PEG-albumin than with HES. PEG-albumin maintained capillary perfusion during the initial stages of hemorrhage and was able to re-establish functional capillaries at the end of hemorrhage. This effect cannot be accounted for by differences in viscosities of both tested solutions, but it might be related to the molecular structure of PEG-albumin. The chemical process of PEGylation adds pseudo-thiols onto the surface of albumin, which increase its binding sites for nitric oxide. Nitric oxide has been shown to be a very important transmitter that regulates vasodilation and therefore influences local blood flow. Our results could be interpreted as the effect of nitric oxide redistribution by PEG-albumin, resulting in improved flow and perfusion pressure to perfuse the capillary network. It has been shown that the number of perfused capillaries is an important predictor for survival from severe hemorrhagic shock [28]. This underlines the importance of the ability of a resuscitation fluid to maintain capillary perfusion. In a setting of resuscitation from severe hemorrhage, one might therefore speculate that pre-hemodilution with PEG-albumin establishes better resuscitation conditions compared with other colloid solutions.

## Key messages

• In the scenario of severe surgical bleeding after preoperative hemodilution, the choice of plasma expander can dictate outcome.

• In our animal model of 50% hemodilution followed by severe 60% blood volume hemorrhage, PEG surface decorated HSA (PEG-albumin; a new type of plasma expander) maintains FCD over time longer than HES 130/0.4.

• The parameter of FCD but not oxygen delivery has been shown to correlate with better outcome in hemorrhagic shock.

• Local perfusion is necessary to limit accumulation of toxic metabolites.

• The chemical process of PEGylation adds pseudo-thiols onto the surface of albumin, which increases its binding sites for nitric oxide. We propose that PEG-albumin increases the transport of nitric oxide to regions of low flow, thereby improving local tissue perfusion.

## Abbreviations

BV = blood volume; FCD = functional capillary density; HAS = human serum albumin; HES = hydroxyethyl starch; HR = heart rate; MAP = mean arterial blood pressure; PaO_2_, arterial oxygen tension; PaCO_2_, arterial carbon dioxide tension; PEG = polyethylene glycol; RBC = red blood cell; TBV = total blood volume.

## Competing interests

PC, SAA and AGT are currently applying for a patent related to the content of this manuscript.

## Authors' contributions

JM participated in the design of the experiments, performed the experiments, and contributed to the manuscript. PC participated in the design of the experiments and the data analysis. KA synthesized the PEG-albumin. SAA synthesized the PEG-albumin and contributed to the manuscript. MI contributed to the manuscript and the data analysis. AGT designed the experiments, performed the statistical analysis, and drafted the manuscript. All authors read and approved the final manuscript.
